# Granulocyte Colony-Stimulating Factor Restored Impaired Spermatogenesis and Fertility in an AML-Chemotherapy Mice Model

**DOI:** 10.3390/ijms222011157

**Published:** 2021-10-15

**Authors:** Yulia Michailov, Ali AbuMadighem, Eitan Lunenfeld, Joseph Kapelushnik, Mahmoud Huleihel

**Affiliations:** 1 The Center of Advanced Research and Education in Reproduction (CARER), The Shraga Segal Department of Microbiology, Immunology, and Genetics, Faculty of Health Sciences, Ben-Gurion University of the Negev, Beer-Sheva 8410501, Israel; yuliadiuro@gmail.com (Y.M.); abumadig@post.bgu.ac.il (A.A.); 2 Barzilai University Medical Center, IVF Unit, Ashkelon 7830604, Israel; 3 The Center of Advanced Research and Education in Reproduction (CARER), Dep OB/GYN, Soroka Medical Center, Faculty of Health Sciences, Ben-Gurion University of the Negev, Beer-Sheva 8410501, Israel; lunenfld@bgu.ac.il; 4 Soroka Medical Center, Department of Pediatric Oncology and Hematology, Beer-Sheva, Faculty of Health Sciences, Ben-Gurion University of the Negev, Beer-Sheva 8410501, Israel; kapelush@bgu.ac.il

**Keywords:** acute myeloid leukemia (AML), male infertility, sperm parameters, testis, granulocyte-colony-stimulating factor (GCSF)

## Abstract

Leukemia and treatment of male patients with anticancer therapy (aggressive chemotherapy and/or radiotherapy) may lead to infertility or even permanent male sterility. Their mechanisms of spermatogenesis impairment and the decrease in male fertility are not yet clear. We showed that under acute myeloid leukemia (AML) conditions, alone and in combination with cytarabine (CYT), there was significant damage in the histology of seminiferous tubules, a significant increase in apoptotic cells of the seminiferous tubules, and a reduction in spermatogonial cells (SALL and PLZF) and in meiotic (CREM) and post-meiotic (ACROSIN) cells. In addition, we showed a significant impairment in sperm parameters and fertilization rates and offspring compared to control. Our results showed a significant decrease in the expression of glial cell line-derived neurotrophic factor (GDNF), macrophage colony-stimulating factor (MCSF) and stem cell factor (SCF) under AML conditions, but not under cytarabine treatment compared to control. In addition, our results showed a significant increase in the pro-inflammatory cytokine interleukin-1 (IL-1) alpha in whole testis homogenates in all treatment groups compared to the control. Increase in IL-1 beta level was shown under AML conditions. We identified for the first time the expression of GCSF receptor (GCSFR) in sperm cells. We showed that GCSF injection in combination with AML and cytarabine (AML + CYT + GCSF) extended the survival of mice for a week (from 6.5 weeks to 7.5 weeks) compared to (AML + CYT). Injection of GCSF to all treated groups (post hoc), showed a significant impact on mice testis weight, improved testis histology, decreased apoptosis and increased expression of pre-meiotic, meiotic and post- meiotic markers, improved sperm parameters, fertility capacity and number of offspring compared to the controls (without GCSF). GCSF significantly improved the spermatogonial niche expressed by increased the expression levels of testicular GDNF, SCF and MCSF growth factors in AML-treated mice and (AML + CYT)-treated mice compared to those groups without GCSF. Furthermore, GCSF decreased the expression levels of the pro-inflammatory cytokine IL-12, but increased the expression of IL-10 in the interstitial compartment compared to the relevant groups without GCSF. Our results show for the first time the capacity of post injection of GCSF into AML- and CYT-treated mice to improve the cellular and biomolecular mechanisms that lead to improve/restore spermatogenesis and male fertility. Thus, post injection of GCSF may assist in the development of future therapeutic strategies to preserve/restore male fertility in cancer patients, specifically in AML patients under chemotherapy treatments.

## 1. Introduction

Acute myeloid leukemia (AML) is a prevalent disease that appears at all ages, but is more frequent in adults. Recently, different studies have shown impairment in sperm parameters among leukemia patients even before anti-cancer treatments [1,2,3,4,5]. Some studies reported a decrease in sperm parameters such as motility, concentration and normal morphology [6,7,8,9,10,11,12], while others reported changes in hormone levels that included a decrease in testosterone and inhibin levels, and an increase in FSH and LH levels [13,14]. Although the precise mechanism has not yet been totally clarified, poor semen quality in these patients may be a result of endocrine/paracrine disturbances, systemic effects of cancer, or both [15,16,17]. Recently, we used an AML adult mice model and showed a significant reduction in testes weight, sperm parameters and fertility within weeks post injection [18].

Leukemia may evoke a systemic response in the body. Cytokines such as interleukins, tumor necrosis factors, and other pro-inflammatory/anti-inflammatory factors secreted by tumor cells and immune system cells may mediate this systemic response [15,16,19,20,21,22,23,24]. Cells of the seminiferous tubules (peritubular cells, Sertoli cells and developed SSCs) showed cell–cell interactions and production of autocrine/paracrine factors that control normal spermatogenesis [25,26,27,28,29]. Stem cell factor (SCF) and its receptor c-kit plays an important role in spermatogonial development [30,31]. Glial cell line-derived neurotrophic factor (GDNF) is secreted by Sertoli and germ cells and is considered an important growth factor for communication between Sertoli cells and spermatogonia [32,33,34]. Macrophage colony-stimulating factor (MCSF or CSF-1) is produced in the testis by Leydig cells, peritubular cells and peritubular macrophages [1,2,3]. Its specific receptor (CSF-1R) has been identified in different cell types, including testicular macrophages, Leydig cells, Sertoli cells, and meiotic cells [4,5,6,7,8,9,10,11,12,13,14,35]. CSF-1 has been shown to directly affect the proliferation of spermatogonial cells and Leydig cell steroidogenesis [13,15,16]. Recently, we demonstrated the involvement of CSF-1 in the induction the proliferation and differentiation of spermatogonial cells to meiotic and postmeiotic stages (BOULE- and ACROSIN-positive cells) [35]. Granulocyte colony-stimulating factor (GCSF) is a glycoprotein that stimulates the bone marrow to produce granulocytes and stem cells and release them into the bloodstream [36]. It is a member of the hematopoietic growth factor family, which regulates the proliferation, differentiation, and survival of hematopoietic progenitor cells [37]. It is produced by endothelium, macrophages, and several other immune cells, and presents in different tissues such as lung, placenta and other tissues. The GCSF has an established safety profile and is successfully used in cancer patients for prevention of chemotherapy-induced neutropenia without decreasing the efficacy of chemotherapeutic agents [37,38]. In the testes, GCSF is produced by Leydig, Sertoli and macrophages [39]. Moreover, the G-CSF receptor CSF3R has been identified in murine SSCs [40]. Recently, it was shown that GCSF treatments in busulfan-treated mice led to significant recovery of spermatogenesis compared to busulfan-treated mice [41]. GCSF appears to promote proliferation of undifferentiated spermatogonia, which leads to a modest enhancement of spermatogenic regeneration from surviving spermatogonia after high-dose busulfan chemotherapy [40]. GCSF also has radio-protective effects on the testicular system. Its injection to male mice three days before gamma irradiation protected about 50% of testicular germ cells from radiation induced apoptosis [42]. Macrophages, which compose around 25% of the interstitial cells [43], may have direct/indirect effects on SSC development. Under physiological conditions, most of the testicular macrophages in rodent are M2 type [43,44]. Macrophages can develop into M1 type, which produces pro-inflammatory cytokines in response to infection/inflammation, while M2 type produces anti-inflammatory cytokines [45,46,47]. Immunoregulatory cytokines have also a fundamental role in spermatogenesis. Indeed, Sertoli cells, peritubular cells and developed SSCs showed production of the IL-1 system (IL-1α, IL-1β, IL-1ra) under physiological conditions [25,26,27,28,29]. These factors are involved in the proliferation/differentiation and apoptosis of SSCs and the functionality of Sertoli and Leydig cells [25,26,27,28,29]. Imbalance in testicular cytokines and growth factors may impair the process of spermatogenesis, thus leading to subfertility/infertility [5]. 

The aim of the present study is to evaluate and deepen our understanding of the possible role and mechanisms of granulocyte colony-stimulating factor in restoration of spermatogenesis and male fertility in AML-chemotherapy treated mice. 

## 2. Results

### 2.1. Localization of GCSF and GCSF-R in Testicular Cells and Spermatozoa, and the Effect of AML and CYT on Their Expression in the Testis

**Figure 1:**
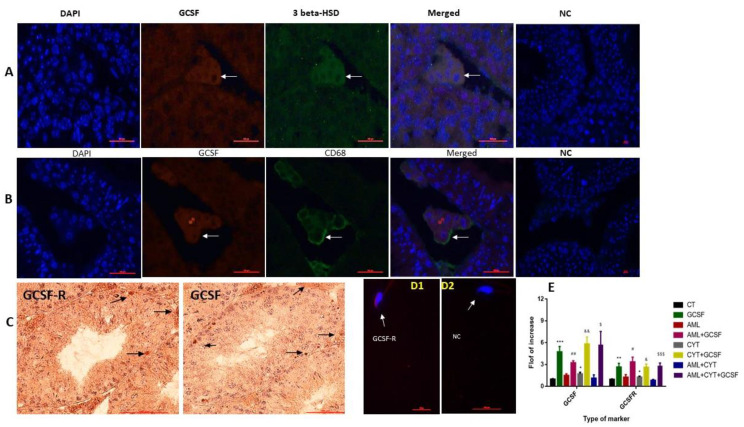
Localization of GCSF and GCSF-R in testicular cells, and the effect of AML and CYT on their expression in the testis. Testicular sections of untreated mice were examined by double immunofluorescence (IF) double staining for GCSF and 3 beta-HSD (as a marker of Leydig cells) (**A**), and for CD68 (a marker of macrophages) (**B**), and the images were merged (orange color). DAPI was also used to show the nucleus of the cells (DAPI; blue staining). Negative control (NC) staining of the tissues was performed as described in the Materials and Methods section and did not show staining of the examined markers. Immunohistochemical (IHC) staining were used to identify the localization of GCSF and its receptor (GCSF-R) in the seminiferous tubules and interstitial of the testis (**C**) by using specific primary antibodies. Negative control (NC) staining of the tissues was performed as described in the Materials and Methods section and did not show staining of the examined markers. In addition, the localization of GCSF-R was examined in the sperm by IF staining using specific antibodies (**D1**) compared to the negative control (NC) (**D2**), without the primary antibodies. DAPI was also used to show the nucleus of the cells (DAPI; blue staining). Arrows indicate the specific cells stained for each marker in the stained tissues. To evaluate the effect of post injection of GCSF on the expression levels of testicular GCSF and its receptor (GCSFR), we used qPCR analysis using specific primers for each factor. qPCR results of each marker are presented as fold of increase compared to the control group (CT) (**E**). The results are representative of 3 independent experiments with 2–3 mice in each group per experiment. *—significant compared to control (CT). #—significant compared to AML, &—significant compared to CYT, $—significant compared to AML + CYT. *,#,&,$—*p* < 0.05; **,##,&&—*p* < 0.01; ***,$$$—*p* < 0.001. Scale bar: 500 µm.

Our results show that GCSF and GCSFR are presented in cells of the interstitial tissue and of the seminiferous tubules (mainly in spermatogonial cells) (Figure 1A–C). Our double immunofluorescence (IF) staining using anti-3- beta HSD expressed in Leydig cells and anti-CD68 expressed in macrophages, demonstrated that both Leydig cells (Figure 1A) and macrophages (Figure 1B) express GCSF in the testis. Moreover, our results also showed the presence of GCSF-R on the head of spermatozoa (Figure 1D1).

Following the above results, we examined the effect of post injection of GCSF on the expression levels of GCSF and GCSF-R in the testes of AML- and CYT-treated mice. Our results show that injection of GCSF or CYT alone or in combination (GCSF + CYT), but not AML or AML + CYT, significantly increased the expression levels of testicular GCSF and GCSF-R compared to control mice (CT) (Figure 1E). However, post injection of GCSF into mice treated with AML (AML + GCSF) or CYT (CYT + GCSF) or AML + CYT (AML + CYT + GCSF) significantly increased the expression levels of testicular GCSF and GCSF compared to AML, CYT or AML + CYT, respectively (Figure 1E). 

### 2.2. Effect of GCSF on the Survival, Testicular Weight and Seminiferous Tubules Histology and Parameters of AML- and CYT-Treated Mice

#### 2.2.1. Mouse Survival

**Figure 2:**
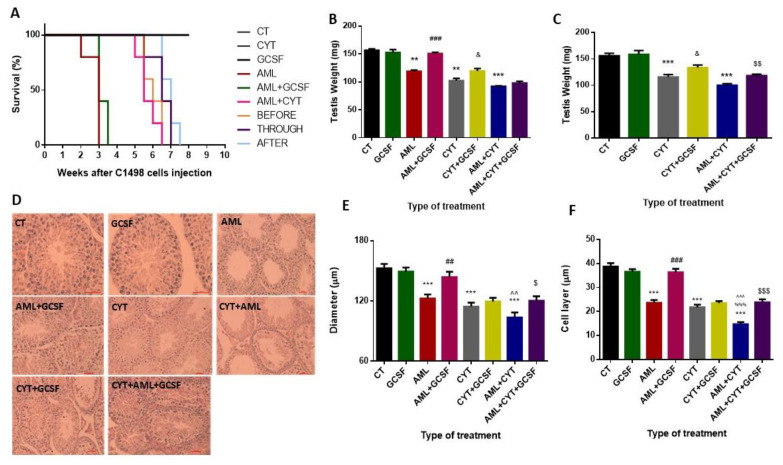
Effect of GCSF on the survival, testicular weight and seminiferous tubules histology and parameters of AML- and CYT-treated mice. Adult C57/black mice were injected with PBS (control, CT), GCSF alone (GCSF), AML cells alone (AML), cytarabine alone (CYT) and in combination with GCSF (AML + GCSF), (CYT + GCSF), combination of both (CYT + AML) and in combination with GCSF (AML + CYT + GCSF) at different injection time points (before injection of AML + CYT, BEFORE; through injection with AML + CYT, THROUGH; and after injection of AML + CYT, AFTER) (**A**). The survival of mice was evaluated 2–8 weeks after injection (see the Methods section) in intervals of one week (**A**). In parallel, the testes of the sacrificed mice were weighted after 3 weeks (**B**) and 5 weeks (**C**) of treatment. Histological analysis using H&E staining (**D**) was used to evaluate the diameters of the seminiferous tubules (**E**) and the cell layer diameters of the seminiferous tubules (**F**) after 3 weeks of treatment. The results are representative of three independent experiments with 5 mice in each group per experiment. *—significant compared to control (CT). #—significant compared to AML, &—significant compared to CYT, $—significant compared to AML + CYT. %—significance of AML + CYT compared to CYT. ^—significance of AML + CYT compared to AML. &,$—*p* < 0.05; **,##,$$^^—*p* < 0.01; ***,$$$,^^^,%%%—*p* < 0.001. Scale bar: 500 µm.

We injected GCSF at three different time points (before, through and after cytarabine treatment) in order to find the most effective time point of injection. Our results show that injection of the mice with PBS (control, CT), GCSF (GCSF) or cytarabine (CYT) did not affect their survival (Figure 2A). Injection of AML cells in combination with GCSF (AML + GCSF), extended mice life from 3 weeks to 3.5 weeks (Figure 2A). Injection of GCSF to the combination group (AML + CYT) before CYT treatment (BEFORE) did not extend the life of the mice (6.5 weeks maximum). However, injection of GCSF following CYT treatment (THROUGH) and after CYT treatment (AFTER) extended mice life from 6.5 weeks (without GCSF; AML + CYT) to 7 and 7.5 weeks, respectively (Figure 2A). Following these results, we chose to inject GCSF, as usually performed by clinicians after (post injection) chemotherapy treatment.

#### 2.2.2. Testis Weight

Our results did not show a significant effect on testicular weight of mice 3 and 5 weeks post injection of GCSF compared to CT (Figure 2B,C, respectively). However, a significant decrease in the testis weight of AML-, CYT- and AML + CYT-treated mice was demonstrated 3 and 5 weeks post treatment compared to CT (Figure 2B,C, respectively). However, post injection of GCSF into AML- (AML + GCSF)-, CYT- (CYT + GCSF) or (AML + CYT)- (AML + CYT + GCSF) treated mice significantly increased their testis weight 3 (except for AML + CYT group) and 5 weeks after the treatments compared to AML, CYT or AML + CYT groups (Figure 2B,C, respectively). 

#### 2.2.3. Seminiferous Tubule Histology (Diameters and Cell Layer)

Our results indicate that 3-week post-injection of GCSF did not affect the histology of the seminiferous tubules (Figure 2D). However, 3-week post-injection of AML cells alone, CYT alone, or AML and CYT in combination (CYT + AML), there was visible damage to tubule integrity and content (Figure 2D). On the other hand, post injection of GCSF into AML- (AML + GCSF), CYT- (CYT + GCSF), or (AML + CYT)- (AML + CYT + GCSF) treated mice showed improvement in the cell layers of the seminiferous tubules compared to the same groups without injection of GCSF (Figure 2D). Quantification of the seminiferous tubules’ diameter and cell layer in the different groups showed that after all treatments (AML, CYT and AML + CYT), there was a significant decrease in tubule diameter compared to CT (Figure 2E,F, respectively). However, 3-week post-injection of GCSF into AML (AML + GCSF) and AML + CYT (AML + CYT + GCSF), but not (CYT (CYT + GCSF), there was a significant increase in the tubule diameter and cell layers compared to AML-, (AML + CYT)- and CYT-treated groups, respectively (Figure 2E,F).

### 2.3. Effect of GCSF on Sperm Parameters, Fertility Capacity and Number of Offspring in AML- and CYT-Treated Groups

**Figure 3:**
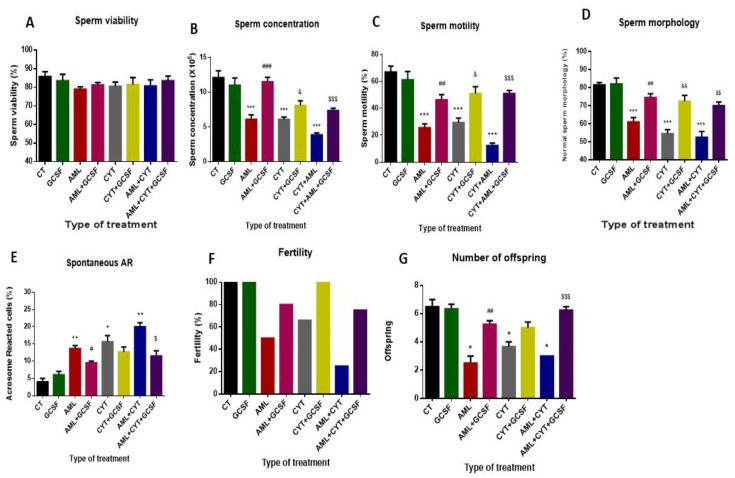
Effect of GCSF on sperm parameters, fertility capacity and number of offspring in AML- and CYT-treated groups. Mice were treated as described in Figure 2. Sperm were extracted from the epididymis 3 weeks post-treatment. Sperm concentration (**A**) was evaluated using a Makler counting chamber and determined according to WHO criteria. Sperm motility/immotility was evaluated using a Makler counting chamber and determined as a percentage of total sperm according to WHO criteria (**B**). Sperm morphology was evaluated following staining with Diff-Quick stain as described previously [18]. Cells were divided into normal and abnormal morphology, which includes: abnormal neck, abnormal tail, abnormal head according to WHO criteria. The percentage of sperm with normal morphology was calculated (**C**). Spontaneous AR was evaluated as described previously [21]. Sperm that were extracted from the epididymis were stained by fluorescein (FITC) staining. Acrosome reacted (without green staining) and non-reacted sperm (with green staining) were counted, and the percent of sperm that underwent spontaneous acrosome reaction was calculated (**D**). Viability of sperm cells was evaluated by their staining with 1% Eosin staining. Dead cells were stained in red color. The percent of live sperm cells was calculated (**E**). To examine the fertility capacity and offspring, two weeks post-treatment, a single male from each group was mated with two females. After two weeks, the females were separated, each to a single cage. The percentage of pregnant females (**F**) and the number of offspring from each female were counted after five weeks (**G**). The results are representative of three independent experiments with 5 mice in each group per experiment. *—significant compared to control (CT). #—significant compared to AML, &—significant compared to CYT, $—significant compared to AML + CYT. *,#,&,$—*p* < 0.05; **,##,&&,$$—*p* < 0.01; ***,###,$$$—*p* < 0.001.

Our results show that 3-week post-injection of GCSF did not significantly affect sperm parameters (viability, concentration, motility, morphology and acrosome reaction), fertility capacity and number of offspring into untreated group (GCSF) compared to control group (CT) (Figure 3A–G, respectively). However, in the AML-, CYT- and (AML + CYT)-treated group, there was a significant reduction in sperm concentration, motility, morphology, fertility capacity and number of offspring, and a significant increase in acrosome reaction, but without a significant effect on sperm viability compared to the control group (CT) (Figure 3A–G, respectively). 

On the other hand, 3-week post-injection of GCSF into AML (AML + GCSF), (CYT (CYT + GCSF) and AML + CYT (AML + CYT + GCSF) significantly increased sperm concentration (Figure 3B), motility (Figure 3C) and morphology (Figure 3D), but decreased spontaneous acrosome reaction (Figure 3E), increased fertility capacity (Figure 3F) and significantly increased the number of offspring (except in CYT + GCSF group) (Figure 3G) compared to AML-, (AML + CYT)- and CYT-treated groups.

### 2.4. Effect of GCSF on Apoptosis of Testicular Cells in AML- and CYT-Treated Groups

**Figure 4:**
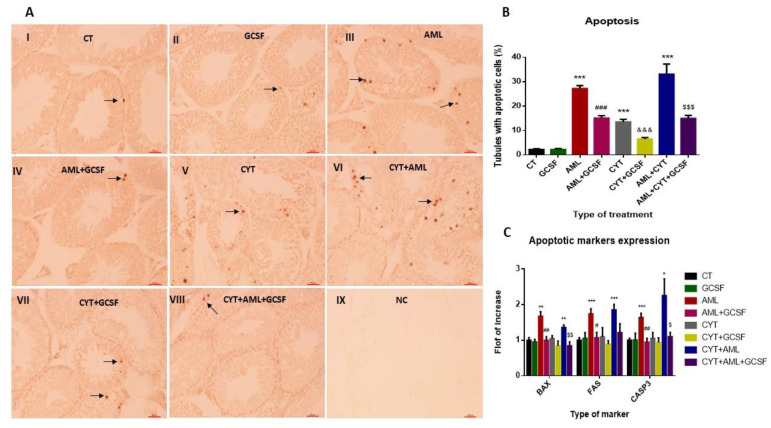
Effect of GCSF on apoptosis of testicular cells in AML- and CYT-treated groups. Mice were treated as described in Figure 2. Testes from mice after 3 weeks of treatment were examined for apoptosis. TUNEL assay (**A**) was performed as described in Materials and Methods for testicular tissue from all the examined groups (control, CT, I; GCSF, II; AML, III; AML + GCSF, IV; CYT, V; CYT + AML, VI; CYT + GCSF, VII; CYT + AML + GCSF, VIII; negative control, NC, IX). The percentage of tubules with more than 5 apoptotic cells has been calculated (**B**). The RNA expression levels of the apoptotic markers (BAX, FAS, Casp3) in testes of all examined groups were tested by qPCR analysis using specific primers (**C**). qPCR results of each apoptotic marker are presented as fold of increase compared to the control group (CT). The results are representative of 3 independent experiments with 3 mice in each group per experiment. *—significant compared to control (CT). #—significant compared to AML, &—significant compared to CYT, $—significant compared to AML + CYT. *,#,&,$—*p* < 0.05; **,##,&&,$$—*p* < 0.01; ***,###,$$$—*p* < 0.001. Scale bar: 500 µm.

Our results show that 3-week post-injection of GCSF had no significant effect on the percentages of seminiferous tubules with apoptotic cells compared to control (CT) (Figure 4A,B). On the other hand, the percentages of seminiferous tubules from AML-, CYT- and (AML + CYT)-treated mice was significantly increased compared to the CT group (Figure 4A,B). However, 3-week post-injection of GCSF into AML (AML + GCSF), CYT (CYT + GCSF) and AML + CYT (AML + CYT + GCSF) significantly decreased the percentages of seminiferous tubules with apoptotic cells compared to AML-, CYT-, (AML + CYT)-treated groups (Figure 4A,B).

We also examined the expression levels of factors involved in the intrinsic (BAX) and extrinsic (FAS and CASP3) pathways of apoptosis in the different treated groups. Our results show that 3-week post-injection of GCSF did not significantly affect the expression levels of both pathways (the BAX, FAS and CASP3 factors) in their testis compared to CT (Figure 4C). On the other hand, in the AML-treated, but not the CYT-treated, group, the expression levels of BAX (intrinsic pathway) and FAS and CASP3 (extrinsic pathway) were significantly increased compared to the control group (Figure 4C). The expression levels of BAX, FAS and CASP3 were significantly increased in (CYT + AML)-treated group compared to CT but not compared to AML group (Figure 4C). However, 3-week post-injection of GCSF into AML- (AML + GCSF) and (AML + CYT)- (AML + CYT + GCSF), but not CYT- (CYT + GCSF), treated group significantly decreased the expression levels of BAX (intrinsic pathway) and FAS and CASP3 (extrinsic pathway) (to become similar to CT group) compared to AML-, (AML + CYT)- and CYT-treated groups (Figure 4C).

### 2.5. Effect of GCSF on the Pre-Meiotic, Meiotic and Post-Meiotic Cells and Their Expression Levels in Testes of AML- and CYT-Treated Groups

**Figure 5:**
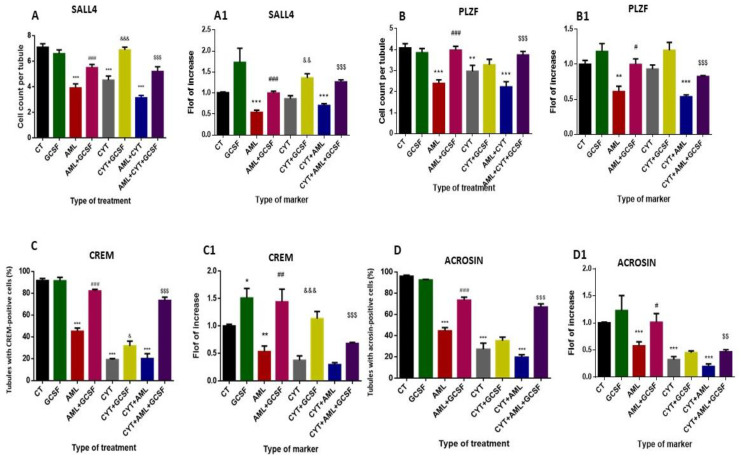
Effect of GCSF on the pre-meiotic, meiotic and post-meiotic cell and their expression levels in testes of AML- and CYT-treated groups. Mice were treated as described in Figure 2. Testicular sections from different groups of treated mice were examined by immunohistochemical (IHC) staining as we described in Figure 1C for the pre-meiotic cell markers (SALL4 and PLZF), for the meiotic marker (CREM) and for the meiotic/post-meiotic marker (ACROSIN) using specific antibodies for each marker. The number of stained cells/seminiferous tubule for SALL4 (**A**), PLZF (**B**) was counted. The percentages of tubules with >10 cells of CREM-positive stained cells (**C**) or ACROSIN-positive stained (**D**) were counted. The RNA expression levels of SALL4 (**A1**), PLZF (**B1**), CREM (**C1**) and ACROSIN (**D1**) in testes of all examined groups were tested by qPCR analysis using specific primers for each marker. qPCR results are presented as fold of increase compared to the control group (CT). The results are representative of 3 independent experiments with 3–5 mice in each group per experiment. *—significant compared to control (CT). #—significant compared to AML, &—significant compared to CYT, $—significant compared to AML + CYT. *,#,&,$—*p* < 0.05; **,##,&&,$$—*p* < 0.01; ***,###,$$$—*p* < 0.001.

Our results show that 3-week post-injection of GCSF into control mice did not significantly affect the percentages and the expression levels of the pre-meiotic (SALL and PLZF), meiotic (CREM) (except the expression levels which increased its expression levels) and the meiotic/post-meiotic cell markers compared to CT (Figure 5). 

#### 2.5.1. Pre-Meiotic Markers 

Our results show a significant decrease in the number/tubule of the pre-meiotic cells SALL4 and PLZF (Figure 5A,B, respectively) (Supplement; Supplement Figure S1A,B; respectively) and in their expression levels (Figure 5A1,B1, respectively) in AML- and CYT-treated mice compared to CT mice. On the other hand, there was no significant effect in the number/tubule and expression levels of SALL4 and PLZF in (AML + CYT)-treated mice compared to the AML- or CYT-treated group, although it was significant compared to CT (Figure 5A,A1,B,B1, respectively). However, with 3-week post-injection of GCSF in AML- (AML + GCSF), CYT- (CYT + GCSF) and (AML + CYT)- (AML + CYT + GCSF) treated mice, there was a significant increase in the number/tubule and expression levels of SALL4 and PLZF compared to AML-, CYT- (except for PLZF; did not change) and (AML + CYT)-treated mice (Figure 5A,A1,B,B1, respectively). 

#### 2.5.2. Meiotic Marker 

Our results show that under normal conditions, more than 90% of seminiferous tubules of the CT group of mice contain more than 10 cells/tubule (tens) that were positively stained to CREM (meiotic cells) (Supplement; Supplement Figure S2) (we arbitrary choose 10 cells/tubule in order to express real change). Additionally, 3-week post-injection of GCSF into untreated CT mice did not significantly affect the percentage of tubules with 10 cells/tubule of CREM-positive cells compared to CT group (Figure 5C), but significantly increased the expression levels of testicular CREM compared to CT group (Figure 5C1). On the other hand, our results show a significant decrease in the percentage of tubules with 10 cells/tubule of CREM-positive cells and CREM expression levels (Figure 5C,C1, respectively) in testicular homogenates of AML- and CYT-treated mice compared to CT mice. However, there was no significant effect in the percentage of tubules with 10 CREM-stained cells/tubule and in the expression levels of CREM in the testicular homogenates of (AML + CYT)-treated mice compared to AML- or CYT-treated group, but it was significantly lower compared to CT (Figure 5C,C1). However, with 3-week post-injection of GCSF into AML- (AML + GCSF), CYT- (CYT + GCSF) and (AML + CYT)- (AML + CYT + GCSF) treated mice, there was a significant increase in the percentage of tubules with 10 CREM-stained cells/tubule and in the expression levels of CREM in their testicular homogenates compared to AML-, CYT- and (AML + CYT)-treated mice (Figure 5C,C1). 

#### 2.5.3. Post-Meiotic Marker 

ACROSIN was used to stain meiotic and post-meiotic cells, including spermatocyte (SPC), round spermatid (RSP) and sperm cells. Our results show that in the control group, almost 100% of tubules contained more than 10 cells/tubule (tens) that were positively stained for ACROSIN (Supplement; Supplement Figure S3) (we arbitrarily chose 10 cells/tubule in order to express real change). Additionally, 3-week post-injection of GCSF into CT mice did not significantly affect the percentage of tubules with 10 cells/tubule of ACROSIN-positive cells or the expression levels of ACROSIN in their testicular homogenates compared to CT group (Figure 5D,D1, respectively). On the other hand, our results show a significant decrease in the percentage of tubules with 10 cells/tubule of ACROSIN-positive cells and ACROSIN expression levels (Figure 5D,D1, respectively) in testicular homogenates of AML- and CYT-treated mice compared to CT mice. However, there was no significant effect in the percentage of tubules with 10 ACROSIN-stained cells/tubule and in the expression levels of ACROSIN in the testicular tissue of (AML + CYT)-treated mice compared to the AML- or CYT-treated group, but it was significant compared to CT (Figure 5D,D1). However, with 3-week post-injection of GCSF into AML- (AML + GCSF), CYT- (CYT + GCSF) and (AML + CYT)- (AML + CYT + GCSF) treated mice there was a significant increase in the percentage of tubules with 10 ACROSIN-stained cells/tubule and in the expression levels of ACROSIN in their testicular tissue compared to AML- and (AML + CYT)-, but not to CYT-treated mice (Figure 5D,D1).

### 2.6. Effect of GCSF on the Expression Levels of Testicular Growth Factors and Pro-Inflammatory Cytokines of AML- and CYT-Treated Mice 

**Figure 6:**
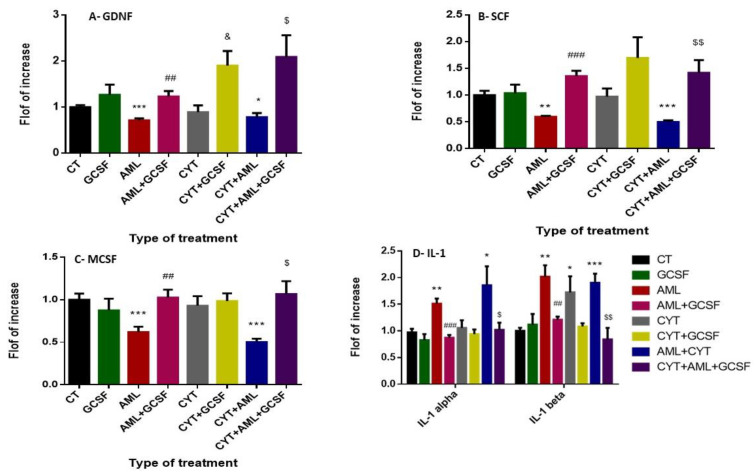
Effect of GCSF on the expression levels of testicular growth factors and pro-inflammatory cytokines of AML- and CYT-treated mice. Mice were treated as described in Figure 2. The RNA expression levels of the growth factors GDNF (**A**), SCF (**B**), MCSF (**C**) and the pro-inflammatory cytokines IL-1 alpha and IL-1 beta (**D**) in testes of all examined groups were tested by qPCR analysis using specific primers for each factor. qPCR results are presented as fold increase compared to the control group (CT). The results are representative of 3 independent experiments with 3 mice in each group per experiment. *—significant compared to control (CT). #—significant compared to AML, &—significant compared to CYT, $—significant compared to AML + CYT. %—significance of AML + CYT compared to CYT. *,#,&,$,%—*p* < 0.05; **,##,&&,$$—*p* < 0.01; ***,###,$$$—*p* < 0.001.

Our results show that 3-week post-injection of GCSF into control mice did not significantly affect the expression levels of GDNF, SCF and MCSF in their testicular homogenates compared to the CT group (Figure 6A–C, respectively). However, a significant decrease in the expression levels of MCSF, GDNF and MCSF in testicular homogenates of AML-treated mice, but not CYT (which was similar to the CT group), compared to control, as examined by qPCR analysis (Figure 6A–C, respectively). On the other hand, we could not detect a significant difference in the expression levels of GDNF, SCF and MCSF in the (AML + CYT)-treated mice compared to the AML-treated mice group, but it was significantly lower compared to CT group (Figure 6A–C, respectively). However, 3-week post-injection of GCSF into AML- (AML + GCSF), CYT- (CYT + GCSF) and (AML + CYT)- (AML + CYT + GCSF) treated mice significantly increased their expression in testicular homogenates compared to AML-, CYT- (except for SCF and MCSF which were similar to CYT-treated group) and (AML + CYT)-treated group (Figure 6A–C).

Our results show that 3-week post-injection of GCSF into control mice did not significantly affect the expression levels of the pro-inflammatory cytokines IL-1alpha and IL-1 beta in their testicular homogenates compared to CT group (Figure 6D). However, a significant increase in the expression levels of IL-1 alpha and IL-1 beta was detected in testicular homogenates of AML-treated mice compared to the CT group (Figure 6D). On the other hand, a significant increase in the expression levels of IL-1 beta but not IL-1 alpha (which was similar to CT) was detected in testicular homogenates of CYT-treated mice compared to the CT group (Figure 6D). Furthermore, the expression levels of IL-1 alpha and IL-1 beta in testicular homogenates of (AML + CYT)-treated group was similar to AML- or CYT-treated group, but were significantly higher compared to the CT group (Figure 6D). On the other hand, 3-week post-injection of GCSF into AML- (AML + GCSF) and (AML + CYT)- (AML + CYT + GCSF), but not CYT- (CYT + GCSF) treated mice (which was similar to CYT group) significantly decreased the expression levels of IL-1 alpha and IL-1 beta in their testicular homogenates compare to AML-, and (AML + CYT)-treated, but not the CYT-treated, group, respectively (Figure 6D).

### 2.7. Effect of GCSF on the Expression Levels of Interstitial Pro-Inflammatory and Anti-Inflammatory Cytokines in AML- and CYT-Treated Mice

**Figure 7:**
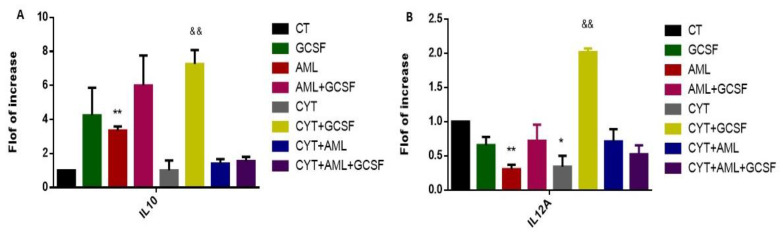
Effect of GCSF on the expression levels of interstitial pro-inflammatory and anti-inflammatory cytokines in AML- and CYT-treated mice. Mice were treated as described in Figure 2. To characterize the type of macrophages present in the interstitial tissue of all examined groups, we evaluated the expression levels of IL-10 (anti-inflammatory cytokine) (**A**) and IL-12A (pro-inflammatory cytokine) (**B**) in the interstitial tissue using qPCR analysis using specific primers for each factor. qPCR results of each marker are presented as fold of increase compared to the control group (CT). The results are representative of 3 independent experiments with 3–6 mice in each group per experiment. *—significant compared to control (CT). #—significant compared to AML, &—significant compared to CYT, $—significant compared to AML + CYT. *,#,&,$—*p* < 0.05; **,##,&&,$$—*p* < 0.01; ***,###,$$$—*p* < 0.001.

To identify the effect of GCSF on the type of active macrophages present in the interstitial of AML- and CYT-treated mice, we tested the ratio between IL-12 and IL-10 expression levels by qPCR analysis, since it is known that M2 macrophages express more IL-10 and less IL-12 and M1 macrophages express more IL-12 and less IL-10. Our results show that AML significantly increased the IL-10 expression levels and significantly decreased the IL-12A expression levels in cells of the interstitial compartment compared to control (Figure 7A,B, respectively). In addition, 3-week post-injection of GCSF alone (GCSF) into the control group or into AML-treated mice (AML + GCSF), significantly increased expression levels of IL-10 but not IL-12A in cells of the testicular interstitial compartment were observed compared to control, but levels in AML + GCSF were similar to GCSF alone (Figure 7A,B, respectively). Additionally, following CYT treatment, there was no significant change in the expression levels of IL-10, but a significant decrease in the expression levels of IL-12A in cells of the interstitial compartment compared to CT (Figure 7A,B, respectively). Injection of GCSF into the CYT-treated group (CYT + GCSF) significantly increased the expression levels of IL-10 and IL-12A compared to the CYT group; however, the fold increase in IL-10 and IL-12 compared to CYT group was similar (around 7 times) (Figure 7A,B, respectively). In addition, the expression levels of IL-10 and IL-12 were similar in the CYT + AML group to those of CYT-treated mice; and post-injection of GCSF (CYT + AML + GCSF) did not significantly affect the expression levels of IL-10 and IL-12 compared to the CYT + AML group. These results may suggest that the M2 macrophages are the main type of macrophages present in the interstitial compartment following the AML and CYT treatment and that post-injection of GCSF did not affect it.

## 3. Discussion

Our results show for the first time the presence of GCSFR on sperm cells. We also showed, in agreement with previous research, the expression of GCSF and GCSFR in cells of the interstitial compartment and in cells of the seminiferous tubules under normal conditions [39,40]. We showed that GCSF was expressed by testicular macrophages and Leydig cells. These findings are in agreement with the results of others, showing GCSF production by Leydig and Sertoli cells in the testis and its presence in the seminal plasma [39]. Our results suggest that GCSF may play a role in the functionality of these testicular cells and in the process of spermatogenesis.

Post-injection of GCSF following AML conditions extended the mouse survival period for a few days. The maximum survival was obtained with injection of GCSF after cytarabine (7.5 weeks survival compared to 6.6 weeks without GCSF). Following these results and knowing that GCSF is used by clinicians after chemotherapy treatment [48], we decided to inject GCSF (i.p) into the mice after the last injection of the chemotherapy. 

We also tested the expression of GCSF and GCSFR in the testes of mice from all treatment groups and found that after GCSF injection to all groups, GCSF and GCSFR expression was significantly increased in their testes. These findings suggest that GCSF regulates its own expression and its receptor in testicular cells. 

The injection of GCSF to all treated groups significantly increased their testes weight 3 and 5 weeks after the AML cell injection compared to AML alone. This was observed in parallel with a decrease in the percentage of the damaged seminiferous tubules (significant increase in tubule diameter and functional cell layer in AML alone and in combination with cytarabine groups following GCSF injection). Additionally, we showed a significant decrease in apoptotic cells in all AML- and/or CYT-treated groups following GCSF injection. GCSF decreased expression of BAX and FAS apoptotic markers that activated both the intrinsic and the extrinsic apoptotic pathways. It is possible that the increase in testicular weight and the decrease in the percentage of tubules with apoptotic cells could be related to the protection against severe damage of the seminiferous tubules following AML disease and chemotherapy treatment. This finding is congruent with other reports demonstrating the prevention of testicular damage from chemotherapy and radiotherapy by GCSF injection [36,37,40,41,42]. Preventing apoptosis by GCSF is mediated by the activation of JAK tyrosine kinases and STAT transcription factors and the ras/mitogen-activated protein kinase pathway [49,50]. In addition, GCSF has an anti-inflammatory effect, as reflected by its down-regulating the production of pro-inflammatory cytokines such as interleukin-6, tumor necrosis factor-alpha, cyclooxygenase-2 and nuclear factor-kappa B in several animal models [50]. GCSF minimizes inflammation, and therefore probably ameliorates the potentially destructive inflammation response. These results provide additional evidence on the mechanisms of the protective effect of GCSF against testicular damage. 

We also examined the possible involvement of GCSF in protecting the spermatogenesis process. After GCSF injection following AML conditions (with or without cytarabine treatment), the pre-meiotic marker PLZF and the post-meiotic marker acrosin quantity and expression were significantly increased compared to the control group. Additionally, the pre-meiotic marker Sall4 and the meiotic marker CREM were significantly increased (quantity and expression levels) following GCSF treatment of the AML- and/or CYT-treated groups. Our results are in agreement with the results of another group that tested the effect of GCSF on spermatogenic regeneration from surviving spermatogonia after busulfan chemotherapy. Normal mice treated with GCSF before or after busulfan treatment exhibited an increase in the numbers of PLZF cells [40]. Our results may explain the increase in testis weight and the improvement of seminiferous tubule histology following GCSF treatment of the study groups. 

Paracrine/autocrine control plays a key role in the regulation of the spermatogenesis process. We demonstrated a decrease in SCF, MCSF, GDNF following AML condition. However, GCSF injection significantly increased the expression levels of SCF, MCSF and GDNF compared to groups without GCSF injection. These findings may suggest that GCSF improved/balanced the testis microenvironment of the study groups, and thus improved spermatogenesis.

In addition, we measured inflammatory conditions (inflammatory cytokines) in the testes all examined groups. We showed that under AML conditions (with or without cytarabine) GCSF injection decreased IL-1 alpha and IL-1 beta expression. These findings may suggest that GCSF may be involved in the regulation of the inflammatory factors in the testes. This is supported by other studies that show the anti-inflammatory abilities of GCSF [42,51,52] 

Due to the protective effect of GCSF in spermatogenesis, we evaluated its effect on the quality of sperm cells in all treated groups. We found that GCSF injection in all treatment groups significantly improved sperm concentration and motility compared to no treatment with GCSF. There was no significant difference in sperm viability between treatment groups. We also showed that GCSF injection improved the fertility capacity of the mice, which was shown by the numbers of offspring. These findings may suggest that GCSF could protect against AML and cytarabine testicular damage, affect sperm production and activity, and, as a result, improve the fertility capacity and eventual number of offspring. 

We showed for the first time the presence and the expression of GCSFR by sperm cells. This may suggest a direct paracrine effect of GCSF in the testes on the functionality of the sperm. Thus, our results may suggest that GCSF may affect spermatogenesis as a paracrine/autocrine factor, while also having a direct effect on the functionality of the developed sperm in the testes under normal and pathological conditions. Injection of GCSF under pathological conditions may restore these functions by increasing the production of GCSF and other paracrine/autocrine growth factors that affect spermatogenic cell development and niches. 

In conclusion, our study used for the first time the AML/CYT-mouse model to demonstrate that AML and CYT may lead to subfertility/infertility. Treatment of these AML/CYT-mice with GCSF enhanced the functionality of the cells in the testes and improved the development of spermatogenesis and fertility capacity. Our study suggests cellular and biomolecular mechanisms for the effectivity of GSCF in restoration and protection of spermatogenesis and fertility capacity in AML- and CYT-treated mice. These results may encourage the development of future therapeutic strategies to preserve male fertility in cancer patients, specifically in AML patients. 

## 4. Materials and Methods

### 4.1. Animals 

This study was performed in accordance with the Guiding Principles for the Care and Use of Research Animals Promulgated by the Society for the Study of Reproduction. It was confirmed by the Ben-Gurion University Ethics Committee for Animal Use in Research (IL-70-11-2016). Six-week-old C57/BLACK mice were purchased from Envigo Laboratories, Jerusalem, Israel. 

### 4.2. C1498/Cell Line and Cytarabine Preparation and Injection

The preparation and intravenously (i.v) injection of the murine C1498 (TIB-49) AML cells were performed according to our previous study [21]. In general, 10^5^ cells/100 µL were injected intravenously per mouse. Cytarabine (CYT, Sigma-Aldrich Israel Ltd., Rehovot, Israel) (100 µL of CYT (3 mg/kg)) was injected intraperitoneally into each mouse. The injections were performed 24 h after the injection of AML cells, 3 times every 12 h (according to Lin, J.M, et al., 2008 [53]; with adaptation). As a control, mice were injected with 100 µL of sterile PBS. 

### 4.3. Mouse Survival 

Survival of mice was examined every week for 8 weeks.

### 4.4. Testis Weight and Other Evaluations

Mice were sacrificed by using Isoflurane (Piramal, PA, USA) and testes were removed from the body and weighed 3 and 5 weeks post treatment. Testes were removed and Bouin fixed and paraffin embedded [54]. 

Hematoxylin and Eosin Staining for Histological Evaluation [54]

The diameter of the testicular tubules was determined by using the mean of vertical and horizontal diameter of each seminiferous tubule (ST). On average, 17 ST were measured from each mouse.

### 4.5. Evaluation of Sperm Parameters

Sperm cells were extracted from the tail of the epididymis by squeezing in a Petri dish plate. Collected sperm from all examined groups of treated mice were examined for concentration and motility using a Makler counting chamber, as described previously [18]. Evaluation of sperm morphology was performed according to the WHO criteria [55], and as described previously [18]. The percentage of acrosome-reacted sperm was determined microscopically on air-dried sperm smears using fluorescein conjugated peanut agglutinin (FITC-PNA, Sigma-Aldrich Israel Ltd., Rehovot, Israel) as described previously [21]. Fertility capacity of mice from all examined groups was determined by mating a single male from each treatment with 2 females (8 weeks old). After two weeks, the females were each separated in different cages. The number of pregnant females and the number of offspring for each female was examined after 4–5 weeks of separation.

### 4.6. Immunohistochemical (IHC) Staining of Testicular Tissue was Performed according to Our Previous Study [54]

Primary and secondary antibodies used are described in Supplement, Table S1. Negative control was performed without the presence of the primary antibodies.

### 4.7. Double Immunofluorescence Staining

Double immunofluorescence staining was performed according to our the method described in our previous study [35,54]. For double immunofluorescence staining of GCSF, specific primary and secondary antibodies that are listed in Supplement, Table S1 were used. 

### 4.8. Evaluation of Apoptosis by TUNEL Assay

TUNEL assay was carried out using a commercial kit (DeadEnd™ Fluorometric TUNEL System, Promega, Madison, WI, USA). The assay was conducted according to the manufacturer’s instructions. TUNEL positive cells were counted using the cell counter and images were acquired using charged coupled device (CCD) camera. The tubules with TUNEL positive cells (more than 5 cells/tubule) were counted and the results are expressed as % of total tubules.

### 4.9. Isolation of Interstitial Cells

The interstitial cells were isolated from the testes of the all-treated groups of mice (3 weeks after injection). Testes were decapsulated and mechanically separated by few accurately aspirations through pipette tips and a 1-mL syringe containing phosphate-buffered saline [PBS, Beit HaEmek Biological Industries (Beit HaEmek, Israel]. The tubules were separated from interstitial tissue by gravity. The suspension of the single cells was filtered through a sterile cell strainer (70 µM; BD Biosciences) and washed with 5 mL PBS (centrifugation at 100× *g* for 5 min). After centrifugation, the media were removed and the pellet of the cells in the bottom of the tube was suspended in 1 mL of PBS.

### 4.10. RNA Extraction and Real-Time Quantitative PCR

This process was performed according to the method in our previous study [54] by using specific primers (Sigma-Aldrich Israel Ltd., Rehovot, Israel). Primer sequences are listed in Supplement, Table S2.

### 4.11. Statistics Analysis

The statistics values were calculated according to Av. ±2 S.D. the statistical significance was examined by two-tailed *t*-test and was shown as: *p*-value < 0.05—*, *p*-value < 0.01—**, *p*-value < 0.001—***.

## Data Availability

The data that support the findings of this study are available from the corresponding author upon reasonable request.

## Supplementary Materials

The following are available online at https://www.mdpi.com/article/10.3390/ijms222011157/s1.
