# Supplementary material for: Granulocyte Colony-Stimulating Factor Restored Impaired Spermatogenesis and Fertility in an AML-Chemotherapy Mice Model
Source: Int J Mol Sci. 2021 Oct 15;22(20):11157. doi: 10.3390/ijms222011157 (PMC8538347; doi:10.3390/ijms222011157)
Supplement: Supplementary file 1 [file ijms-22-11157-s001.zip › ijms-1404420-supplementary.pdf]

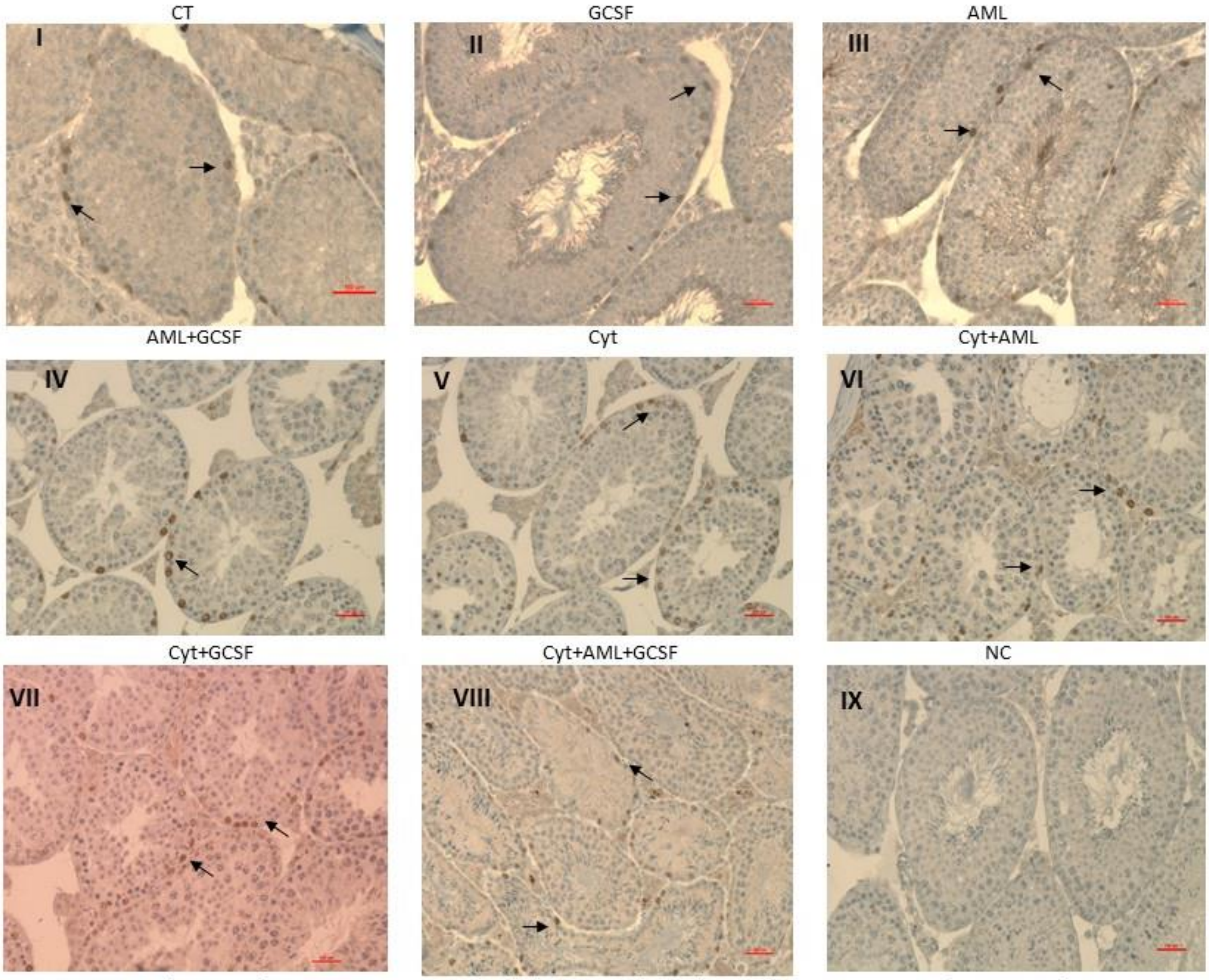

Figure S1A. Localization of SALL4-positive stained cells by immunohistochemical staining in testicular sections from mice with different treatments.

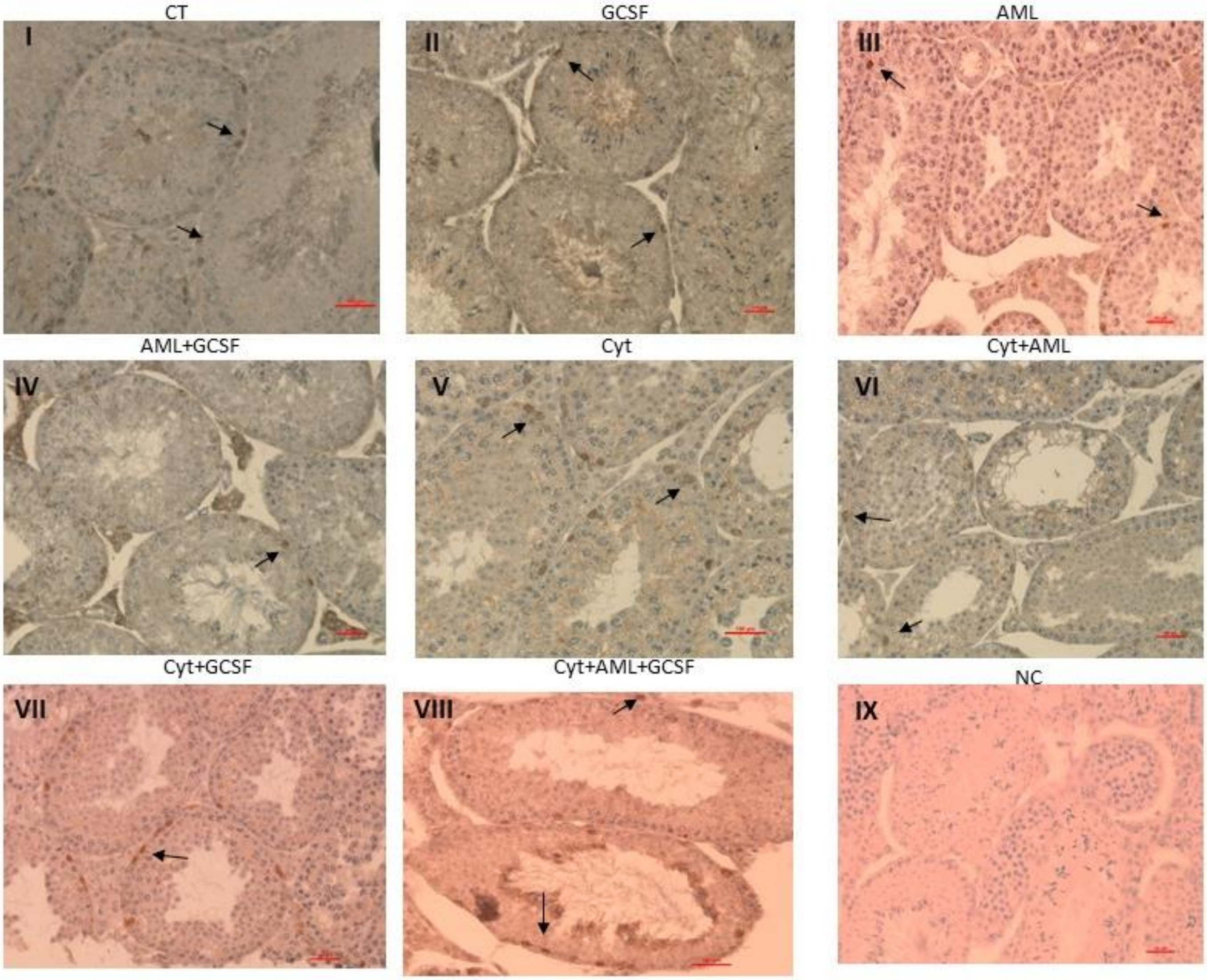

<u>Figure S1B.</u> Localization of PLZF-positive stained cells by immunohistochemical staining in testicular sections from mice with different treatments.

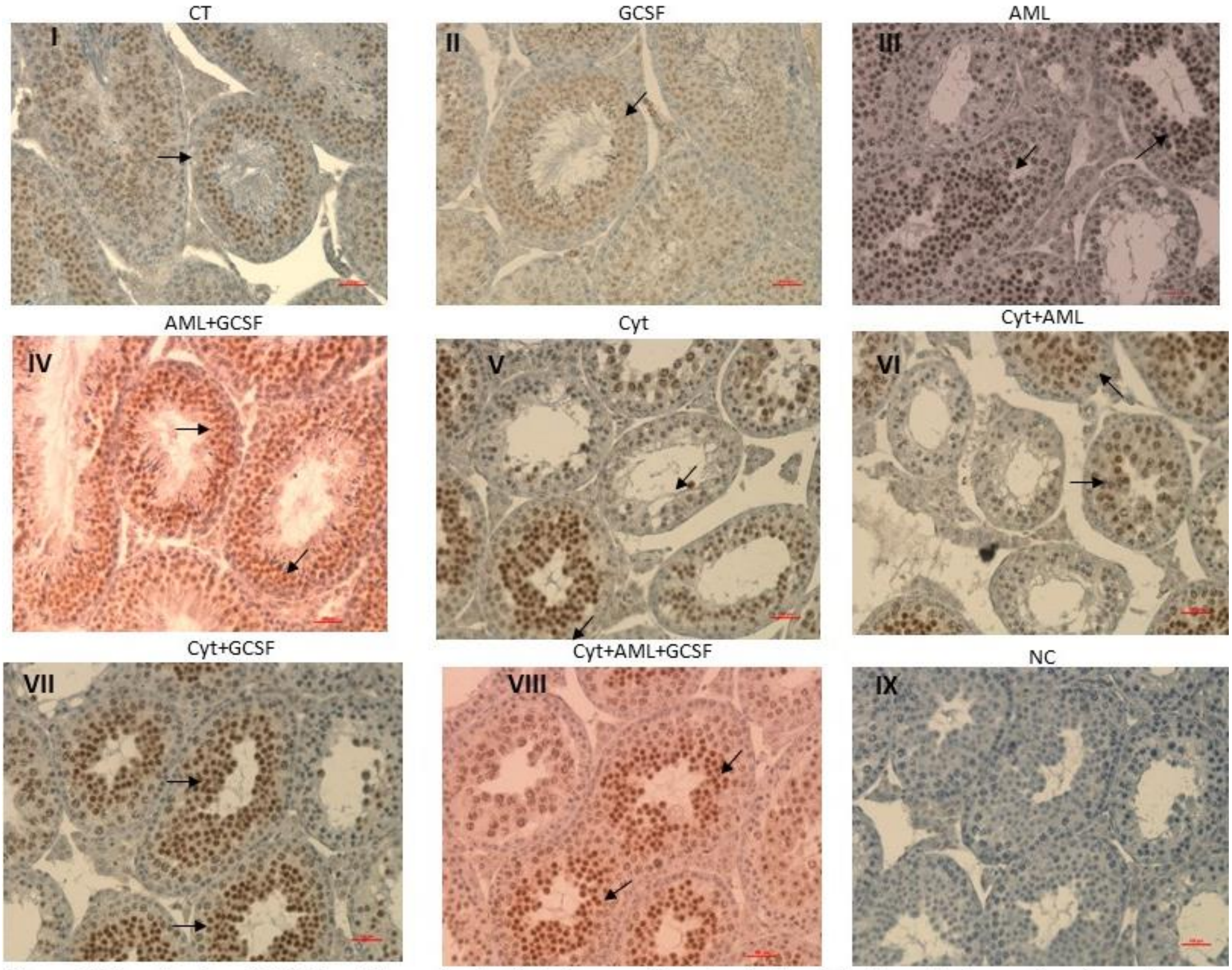

Figure S2. Localization of CREM-positive stained cells by immunohistochemical staining in testicular sections from mice with different treatments.

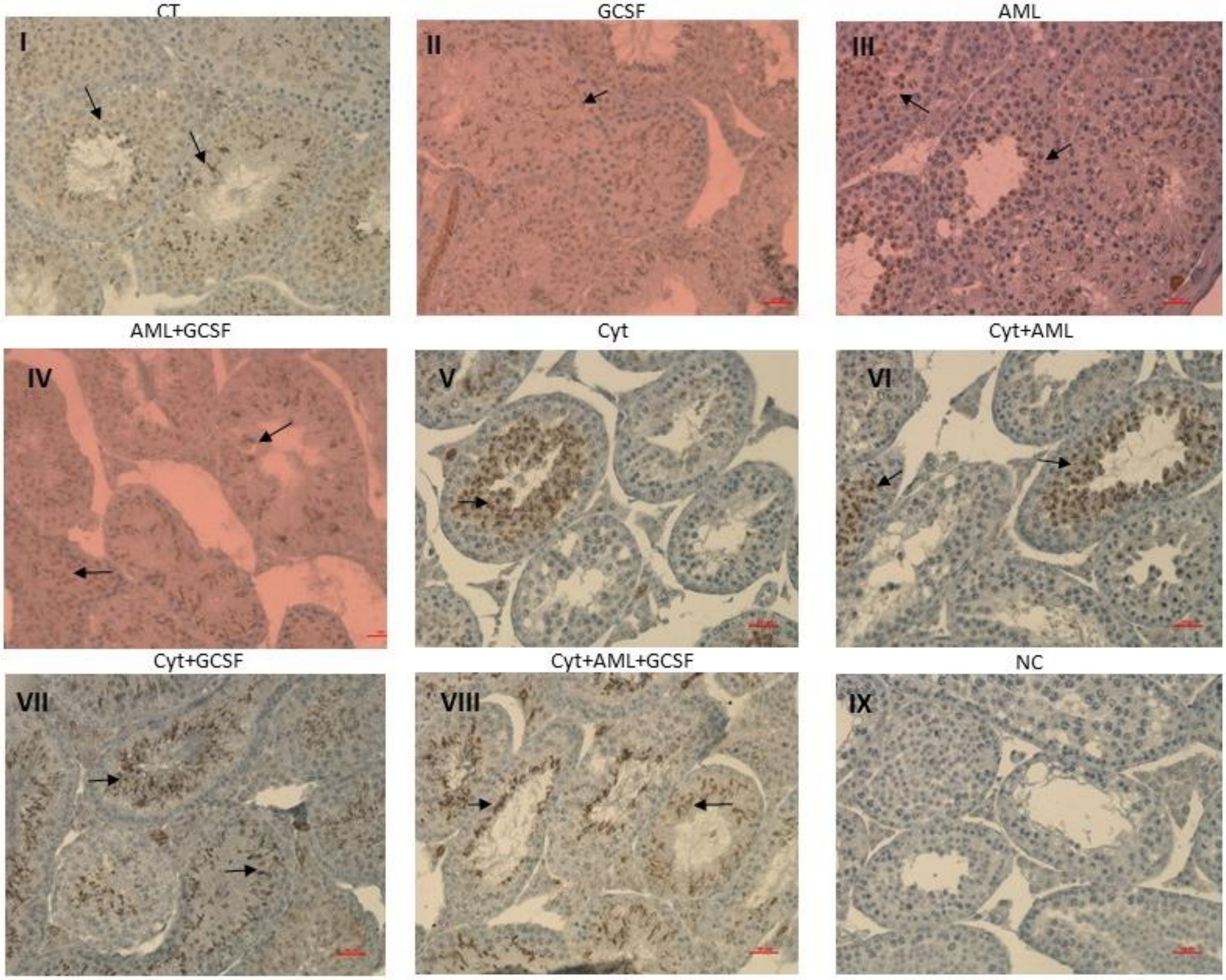

<u>Figure S3.</u> Localization of ACROSIN-positive stained cells by immunohistochemical staining in testicular sections from mice with different treatments.

Table .S1

Primary antibody	Secondary antibody
Polyclonal rabbit anti-mouse PLZF, Santa Cruz, CA, USA	horse anti-rabbit IgG, Vector, CA, USA
Polyclonal rabbit anti-mouse SALL4, Abcam, Cambridge, MA,USA	horse anti-rabbit IgG, Vector, CA, USA
Polyclonal rabbit anti-mouse CREM, Santa Cruz, CA, USA	horse anti-rabbit IgG, Vector, CA, USA
Polyclonal rabbit anti-mouse ACROSIN, Novus Biologicals, CA, USA	horse anti-rabbit IgG, Vector, CA, USA
Polyclonal rabbit anti-mouse GCSF, Santa cruz (Santa Cruz, CA, USA)	Donkey anti rabbit IgG (Rhodamine red), Jackson Immuno Research (West Grove, PA USA)
Polyclonal rabbit anti-mouse GCSF-R, Bioss (Boston,USA)	Donkey anti rabbit IgG (Rhodamine red), Jackson Immuno Research (West Grove, PA USA)
Polyclonal goat anti-mouse 3β HSD, Santa cruz (Santa Cruz, CA, USA)	Donkey anti goat IgG (Cy3), Jackson Immuno Research (West Grove, PA USA)
Monoclonal mouse anti-mouse CD68, Santa cruz (Santa Cruz, CA, USA)	Goat anti mouse IgG (Rhodamine red), Jackson Immuno Research (West Grove, PA USA)

Table .S2

Gene	Forward	Reverse
PLZF	AGCTTGAAATACGTGGCCAGA	TGAGCAGTTCACACTTCATCCC
SALL4	GAAAGCCCACAATTTCTCCTG	AGGAAACAGGCAGTTTTCCAA
CREM	TTCTTTCACGAAGACCCTCA	TGTTAGGTGGTGTCCCTTCT
ACROSIN	TGTCCGTGGTTGCCAAGGATAACA	AATCCGGGTACCTGCTTGTGAGTT
BAX	GAGAGGTCTTCTTCCGGGTG	CTTGGATCCAGACAAGCAGC
FAS	GCTGGCTCACAGTTAAGAGTT	GTTGGTGTACCCCCATTCATT
CASP3	TCATCTCGCTCTGGTACGGA	ACACACACAAAGCTGCTCCT
GAPDH	ACCACAGTCCATGCCATCAC	CACCACCCTGTTGCTGTAGCC
SCF	TGAGCCCTTATGCCACACAAT	AAGATGATCCCAAACGCTCGT
MCSF	CCCATATTGCGACACCGAA	AAGCAGTAACTGAGCAACGGG
GDNF	GCCCCTGCTTTCTATCTGCT	AGCCTTCTGAATGCGTGGTT
IL1-α	GAAGCTCGTCAGGCAGAAGT	GTGCACCCGACTTTGTTCTT
IL1-β	CAGGATGAGGCATGAGCACC	CTCTGCAGACTCAAACTCCAC
IL-10	CGGGAAGACAATAACTGCACCC	CGGTTAGCAGTATGTTGTCCAGC
IL-12A	TCTTTGATGATGACCCTGTGC	TCTCCCACAGGAGGTTTCTG
